# A short-term inhalation study to assess the reversibility of sensory irritation in human volunteers

**DOI:** 10.1007/s00204-020-02703-8

**Published:** 2020-03-17

**Authors:** Stefan Kleinbeck, Michael Schäper, Marlene Pacharra, Marie Louise Lehmann, Klaus Golka, Meinolf Blaszkewicz, Thomas Brüning, Christoph van Thriel

**Affiliations:** 1 Leibniz Research Center for Working Environment and Human Factors, TU Dortmund University, Ardeystr. 67, 44139 Dortmund, Germany; 2 MSH Medical School Hamburg, University of Applied Sciences and Medical University, Am Kaiserkai 1, 20457 Hamburg, Germany; 3 Charité, Universitätsmedizin Berlin, Augustenburger Platz 1, 13353 Berlin, Germany; 4 Research Institute for Prevention and Occupational Medicine of the German Social Accident Insurance, Institute of the Ruhr University Bochum (IPA), Bürkle de la Camp-Platz 1, 44789 Bochum, Germany

**Keywords:** Sensory irritation, 5-Day controlled exposure study, Objective measures of irritation, Eye blinking frequencies, Perceptual ratings, Ethyl acrylate, Biochemical markers of neurogenic inflammation

## Abstract

Sensory irritation is an acute adverse effect caused by chemicals that stimulate chemoreceptors of the upper respiratory tract or the mucous membranes of the outer eye. The avoidance of this end point is of uttermost importance in regulatory toxicology. In this study, repeated exposures to ethyl acrylate were analyzed to investigate possible carryover effects from day to day for different markers of sensory irritation. Thirty healthy subjects were exposed for 4 h on five subsequent days to ethyl acrylate at concentrations permitted by the German occupational exposure limit at the time of study. Ratings of eye irritation as well as eye blinking frequencies indicate the elicitation of sensory irritation. These markers of sensory irritation showed a distinct time course on every single day. However, cumulative carryover effects could not be identified across the week for any marker. The rhinological and biochemical markers could not reveal hints for more pronounced sensory irritation. Neither increased markers of neurogenic inflammation nor markers of immune response could be identified. Furthermore, the performance on neurobehavioral tests was not affected by ethyl acrylate and despite the strong odor of ethyl acrylate the participants improved their performances from day to day. While the affected physiological marker, the increased eye blinking frequency stays roughly on the same level across the week, subjective markers like perception of eye irritation decrease slightly from day to day though the temporal pattern of, i.e., eye irritation perception stays the same on each day. A hypothetical model of eye irritation time course derived from PK/PD modeling of the rabbit eye could explain the within-day time course of eye irritation ratings repeatedly found in this study more precisely.

## Introduction

Occupational exposure limits (OELs) are thought to protect workers from acute and chronic health effects related to the chemicals they are exposed to (ACGIH 2019; DFG 2018). Among the acute effects of volatile chemicals, the avoidance of sensory irritation is of high relevance in the working environment (Brüning et al. 2014; Nielsen and Wolkoff 2017). This critical end point was introduced and conceptually described by Yves Alarie in the early 1970s (Alarie 1973). Due to the activation of chemoreceptors/nociceptors (e.g., transient receptor potential channels; TRP channels) located in the membrane of free endings of peripheral nerves innervating the airways (Bessac and Jordt 2008), various reflexes or defense mechanisms can be elicited by particular chemicals, so-called sensory irritants (Schaper 1993). To estimate the potency of a chemical to cause sensory irritation, a bioassay has been proposed (Alarie 1981). Here, a standardized inhalation procedure in rodents is used to determine the concentration of a chemical that decreases the respiration rate of the animals to 50% of the baseline (RD_50_). Since then the respective RD_50_ was related to many OELs (Nielsen et al. 2007; Schaper 1993), indicating a systematic relationship (OEL ~ 0.03 × RD_50_). However, this relationship was mainly based on comparisons among animal studies (Bos et al. 2002) and the extrapolation to humans is still difficult due to anatomical and physiological species differences of the upper airways (Brüning et al. 2014). In case of ethyl acrylate, an RD_50_ of 315 ppm has been reported (de Ceaurriz et al. 1981) which would lead to a tentative OEL of almost 10 ppm that is still higher than the NOAEC derived from human exposure studies (Kleinbeck et al. 2017; Sucker et al. 2019).

If such human data are available, it is assumed that the avoidance of physiological indicators of sensory irritation, such as trigeminal-mediated reflexes (e.g., increase of eye blinking frequency) or neurogenic inflammation (e.g., release of neuropeptides such as substance P), also protects the organism from chronic health effects such as tissue irritation (Brüning et al. 2014). Information about such chronic effects of exposure to irritants is usually obtained in sub-chronic or chronic inhalation studies in rodents. For ethyl acrylate, such chronic inhalation studies (6 h/day, 5 days/week, up to 27 months) of rats and mice revealed a lowest observed effect concentration (LOAEC) of 25 ppm and no observed effect concentration (NOAEC) of 5 ppm based on non-neoplastic changes at the olfactory epithelium, hyperplasia, and inflammation of the Bowman glands (Miller et al. 1985). Due to the lower intranasal metabolism of ethyl acrylate in humans as well as a valid NOAEC in rodents, workers should be protected to suffer from tissue irritation at an OEL of 5 ppm (Brüning et al. 2014).

This assumption is not only based on empirical comparisons of substances with available data, but also neurobiological mechanisms underlying sensory irritation can be taken into account. Various chemoreceptors expressed on the free nerve endings of trigeminal fibers in the nose and the eyes can be activated by various chemicals (Lehmann et al. 2017), leading to specific perceptions (e.g., burning, stinging; cf. Hummel 2000) and various reflexes and defense mechanisms such as sneezing or lacrimation. This response cascade, underlying the selection of end points and effect markers in experimental exposure studies with human volunteers (Brüning et al. 2014), has recently been structured according to the concept of the adverse outcome pathway (AOP; Ankley et al. 2010). Martinez and Eling (2019) describe such an AOP for sensory irritation starting with the activation of the transient receptor potential ankyrin 1 (TRPA1) ion channel by volatile organic chemicals (VOCs) as molecular-initiating event (MIE). Based on the review of relevant literature, they described three key events (KE) within the peripheral nervous system that via neurogenic inflammation can cause the adverse outcome (AO) of sensory irritation. Based on these neurobiological considerations, it is further assumed that up to a certain threshold these responses of the peripheral nervous system are completely reversible. However, due to neuronal plasticity and interactions with the immune system (Chiu et al. 2012), exposures above such thresholds might lead to sensitization. Here, repeated exposures to sensory irritants might be crucial, since recovery periods are required to somehow “reset” this response cascade. These aspects are not considered in the AOP by Martinez and Eling (2019).

In the context of human health risk assessment, experimental exposure studies with human volunteers are considered to be the gold standard for the derivation of No Observable Adverse Effect Concentrations (NOAECs) for sensory irritation (Brüning et al. 2014; Nielsen and Wolkoff 2017). In most controlled human exposure studies, there are exposure-free time periods between various sessions to avoid carryover effects and to evaluate different concentrations independently. In contrast to these experimental studies, the regulation of chemicals assumes that workers are usually exposed to irritants their whole working life on the basis of five days per week. As mentioned previously, two of such studies (Kleinbeck et al. 2017; Sucker et al. 2019) report sensory irritation as measured by eye blinking frequencies in human subjects during a single 4-h exposure with constant or varying exposure to 5 ppm of ethyl acrylate. Though 5 ppm of ethyl acrylate proved to be a NOAEC in chronic animal studies, it remains unclear whether the same is true for human subjects exposed in a similar manner (i.e., repeated exposure). Ethyl acrylate was chosen as model compound as there is a good database (Brüning et al. 2014; Kleinbeck et al. 2017) and, therefore, an extrapolation/read-across to other sensory irritants might be possible. Inspired by the procedure of short-term inhalation studies (5 days) in rodents using nano-seized materials (cf. Ma-Hock et al. 2007), a controlled 5-day exposure study with human volunteers was conducted simulating the exposure of a working week with chemical exposure to evaluate whether the interspecies extrapolation is appropriate in the case of ethyl acrylate.

Based on the results from our previous experimental exposure with healthy volunteers (Kleinbeck et al. 2017), we here investigate the question if exposures to such irritating concentrations (human LOEAC) may exceed the threshold of sensitization under common working conditions (exposure on subsequent days) or whether the exposure effect is completely reversible. Therefore, this study was designed to discover possible carryover effects of sensory irritation in humans and to replicate the main results of Kleinbeck et al. (2017).

## Materials and methods

### Subjects

Thirty healthy non-smoking male (*n* = 14) and female (*n* = 16) subjects aged 19–35 years (mean: 25 years) participated in this study. The participants were recruited at the Technical University of Dortmund. They were examined by a physician during a training session. The medical examination included clinical blood chemistry tests, an electrocardiogram (ECG), and a test of lung function. In cases of chronic diseases of respiratory tracts, suspicion of other diseases (e.g., hypertonia and liver dysfunctions), or neurological dysfunctions (e.g., head trauma), subjects were excluded. On this occasion, subjects were also trained in the handling of methods used during the study. The study protocol was approved by the ethics committee of the Leibniz Research Centre for Working Environment and Human Factors at TU Dortmund University. Furthermore, written informed consent was obtained prior to the experiments. Sniffin’ Sticks (Hummel et al. 1997) were used to assess subjects’ olfactory ability. Based on normative data (Hummel et al. 2007), hyposmic subjects were excluded.

### Experimental exposure

The study was conducted in the exposure laboratory of the Leibniz Research Centre for Working Environment and Human Factors at TU Dortmund University (cf. Pacharra et al. 2016). The laboratory, comprising a secluded room of 29 m^3^, is made of stainless steel and glass windows. There are four workplaces in the laboratory and, therefore, four subjects were exposed simultaneously. The workplaces were equipped with 15 in. color computer monitors. Ratings and neurobehavioral testing were administered at these workplaces. The laboratory was aerated by a climate control unit located in an adjacent room. A heater platform was used to vaporize ethyl acrylate. Vaporized ethyl acrylate was brought into the inlet airflow. The conditioned air was delivered to the laboratory by a branched pipe system on the floor. Exhaustion of the laboratory was accomplished by four outlets located at the ceiling. A contamination of the surrounding laboratory in case of leakage was avoided by a negative pressure between 20 and 30 Pa maintained in the laboratory. The average air exchange rate was 300 m^3^/h. The airborne concentration of ethyl acrylate was monitored by four sampling devices located at the ceiling of the laboratory. Samples were taken every 80 s maintaining a quasi-continuous monitoring and were analyzed by photoacoustic IR spectrometry (INNOVA, 1412 Photo Acoustic Field-Gas-Monitor; Photo Acoustic Detector PAD). The results were monitored online and stored on hard disk for subsequent analyses.

**Fig. 1:**
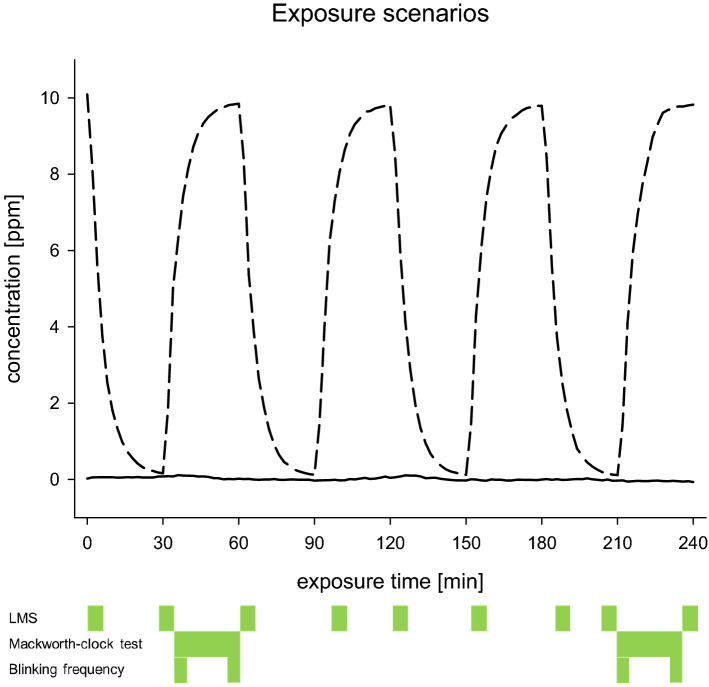
Time courses of the measured ethyl acrylate concentration during the two conditions (0 ppm and 0–10 ppm) together with times of LMS ratings and eye blinking frequency measures. The solid line indicates the control condition (0 ppm), and the dashed line the varying condition (0–10 ppm)

Two 4 h exposure scenarios were used. An experimental condition, corresponding to the higher varying condition (0–10 ppm) in Kleinbeck et al. (2017), was investigated as this condition led to the most pronounced chemosensory effects. This exposure corresponds to the German OEL (5 ppm TWA with a STEL of 10 ppm) at the time the experiment was conducted (2012) following the recommendation of the MAK-Commission (MAK 2007) and of the Scientific Committee on Occupational Exposure Limits (SCOEL 2004). During a control condition, there was clean air (0 ppm ethyl acrylate) in the laboratory (see Fig. 1). Subjects were exposed to each condition on five subsequent exposure days (Monday–Friday). Exposure weeks were separated by 1 week without exposure.

The chemical analytic data revealed that the targeted concentrations were achieved with high precision and accuracy: *C*_TWA_ = 5.01 ppm, *C*_min_ = 0.11 ppm, *C*_max_ = 10.1 ppm.

A repeated-measurement crossover design was used. Half of the subjects started with the control condition (0 ppm), while the other half started with the experimental condition (0–10 ppm).

### Assessment of dependent variables

#### Ratings of intensity of chemosensory sensations

Chemosensory effects of ethyl acrylate were assessed by a visual analog scale (VAS) using the format of the ‘labeled magnitude scale’ (LMS; Green et al. 1996) that mimics the ratio-like properties of magnitude estimation scaling (Green et al. 1996). LMS is a widely used scale to rate the intensity of chemosensory stimuli (Hey et al. 2009; Juran et al. 2012; Kleinbeck et al. 2008, 2017; van Thriel et al. 2005, 2007a, 2010).

Olfactory and trigeminal descriptors were used to rate different perceptions: odor intensity, annoyance, and nauseous as olfactory perceptions; and eye irritation, burning, tickling, nasal irritation, ‘sneeze’, prickling, sharp, and pungent as trigeminal perceptions (Laska et al. 1997).

The LMS rating was administered via PC workplaces in the laboratory. The scale label was shown on the top of the screen (e.g., odor intensity). A slider on the left side and six categories (ranging from barely detectable to strongest imaginable, numeric range: 1–1000) close to the slider allow for rating of the intensity of the sensation given above. Subjects could move a small arrow to that position that corresponds to the intensity of their current sensation with the help of a sliding controller. The ratings were conducted approximately every 30 min during exposure starting when entering the exposure laboratory (cf. Fig. 1). A pre- and post-rating were conducted 1 h before the exposure started and about 55 min after the end of exposure in clean air.

#### Physiological measures

Standardized recordings of eye blinks were performed at fixed times during each exposure (Kiesswetter et al. 2005). As eye blinks are caused by contractions of the orbicularis oculi muscle, an electromyography of this particular muscle was used to count the closings of the eyelid. To standardize visual demands, assessments of eye blinking frequencies were executed during a vigilance task that was applied in the format of the Mackworth-clock test (Parasuraman et al. 2000). This test was conducted two times during exposure starting at minutes 35 and 210, respectively. The vigilance task had a duration of 25 min and for statistical analysis only the first and last 5 min of the tasks were selected for assessment of eye blinking frequency. This selection was done to be able to compare the low exposure with the high exposure, as both Mackworth-clock tests were conducted in an ascending slope of concentration during the varying conditions. Comparable analyses have been conducted before (Kiesswetter et al. 2005; Kleinbeck et al. 2008, 2017). Measuring eye blinking frequencies allows for a comparison of objective (eye blinking frequencies) measures and subjective ratings (of eye irritation).

To measure nasal airway resistance quantitatively, anterior active rhinomanometry (AAR) was used. Nasal congestion which might have been induced by sensory irritation in the nose caused by ethyl acrylate exposure would have lowered the nasal airflow. A computer-based system (Atmos Inc., Lenzkirch, Germany) was used to measure the transnasal pressure gradient and nasal flow. AAR was determined before and after exposure on each exposure day to identify changes in nasal flow (∆Flow) caused by the exposure to ethyl acrylate.

Additionally, nasal lavages were performed before and after the exposure on each exposure day. A sterile pipette (NUNC™) was filled with 10 ml of phosphate-buffered saline (PBS; pre-warmed at 36 °C) and 5 ml of the PBS was filled into each nasal cavity. The PBS remained in the nose for 10 s. After that, the subjects let the lavage fluid flow into funneled 15 ml PP-test tubes without sniffing. The volume of the lavage fluid recovered after the nasal lavage was recorded. The lavage samples were stored on ice immediately and were frozen at − 80 °C within the next 30 min. No protease inhibitors were added, as substance P concentration in nasal lavage fluid stored at 4 °C proved to be stable up to 50 h without any additives (Schultz et al. 1996). Substance P in nasal lavage fluid as an indicator of neurogenic inflammation (e.g., Hunter et al. 2000; Nikasinovic-Fournier et al. 2002) was analyzed by means of ELISA kits from Cayman Chemical (Ann Arbor, MI, USA; local dealer: IBL-Hamburg, Nr. CM59211). Additionally, tumor necrosis factor alpha (TNF alpha) and interleukin-8 (IL-8) in nasal lavage fluid as an indicator of immune system activity (Chiu et al. 2012) were analyzed by means of ELISA kits.

#### Neurobehavioral tests

Several neurobehavioral tests were conducted during exposure: two-back tests (Kirchner 1958) with objects and with spatial positions, a divided attention task (Zimmermann and Fimm 1993), a modified flanker task (Carbonnell and Falkenstein 2006), and the Mackworth-clock test (Parasuraman et al. 2000) mentioned above.

### Statistics

For the LMS data, repeated-measures analyses of variance (ANOVAs) were carried out for each perception with condition/concentration (two conditions; factor name: *condition*) as a within-subjects factor. Due to different interesting temporal resolutions, two temporal factors were included: across-days factor (five subsequent days representing changes from day to day; factor name: *day*) and within-day factor (nine repeated measures representing duration and concentration changes during exposure; factor name: *time*), both as within-subject factors. The interaction of both temporal factors is of special interest, as it could reveal subtle carryover effects from day to day. The factor sex is included as a between-subjects factor.

Eye blinking frequency was analyzed by means of a repeated-measurements ANOVA with condition/concentration (*condition*) and across-days factor (*day*) as within-subject factor. For the statistical analysis of the eye blinking frequency, the second within-day factor *time* (4 repeated measures for this readout; cf. Fig. 1) was split into two nested factors (cf. Kleinbeck et al. 2017): *exposure duration* (first and second Mackworth-clock test, comprising two measures each; cf. Fig. 1), and *current concentration* (first and last 5 min periods of the 25 min vigilance task corresponding to low and high concentrations during the varying conditions; cf. Fig. 1), both as within-subject factors. Sex was added as a between-subjects factor.

Neurobehavioral tests were analyzed with repeated-measures ANOVA regarding the factor concentration, across-days factor (day) and within-day factor (time) as within-subject factors. The task-related factors were complemented depending on the task, e.g., compatibility was used to describe the response-inhibition (flanker task) results, to complete the analysis of the neurobehavioral tasks. Additionally, the between-subject factor sex was included.

Differences between pre- and post-measures in active anterior rhinomanometry were analyzed by a two-factorial repeated measures ANOVA (pre–post measurement, 2 conditions, 5 days).

For substance P and 15-HETE, differences between pre- and post-measures were analyzed by a Friedman test for the two conditions on each day.

All statistical analyses were conducted with IBM SPSS Statistics 25. A significance level of *p* ≤ 0.05 was used. In case of violation of the assumption of sphericity, Greenhouse–Geisser corrected degrees of freedom were used.

## Results

Since this study addressed two different aims, namely (a) the replication of previous results obtained for ethyl acrylate (Kleinbeck et al. 2017) and (b) the investigation of possible temporal summation of sensory irritation across a simulated 5-day working week, the presentation of the results section is structured accordingly.

### Replication of results from a previous volunteer study

**Fig. 2:**
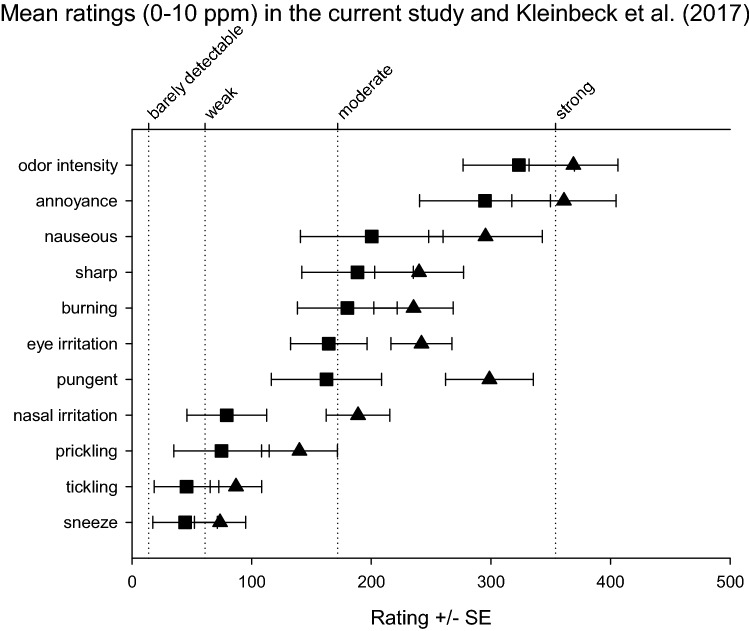
Mean ratings during the 0–10 ppm exposure condition in the current study (triangles) and in Kleinbeck et al. (2017) (squares)

In comparison to the similar exposure scenario (0–10 ppm) used in the experiments reported by Kleinbeck et al. (2017), the first exposure day (Monday) of this study revealed an almost similar pattern of intensity ratings of the olfactory and trigeminal perceptions (cf. Fig. 2).

Volunteers of the current study reported more intense perceptions with pronounced differences for eye irritation, pungent, and nasal irritation ratings. This might be partly caused by the other exposure conditions tested in the previous study. Thus, in contrast to this study, the particular 0–10 ppm condition was not the first experience with ethyl acrylate for all subjects.

**Fig. 3:**
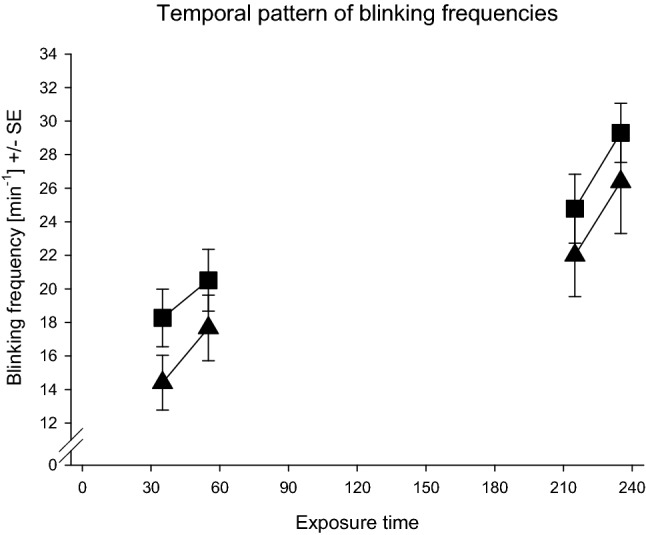
Temporal pattern of eye blinking frequencies during the 0–10 ppm exposure condition in the current study (triangles) and in Kleinbeck et al. (2017) (squares)

The time course of the eye blinking frequencies (cf. Fig. 3) of the current study was on a lower level.

Nevertheless, exactly the same temporal pattern across the 4-h exposure period was observed. In both experiments, total exposure duration and the concentration peak increased the eye blinking frequencies, a valid physiological indicator of sensory irritation, of the volunteers. Again, and confirming the results of the previous study, none of the other investigated physiological parameters was affected by the 0–10 ppm exposure.

After showing the good reproducibility of our previous results, we analyzed the effects of repeated exposures to this particular exposure scenario.

### Sensitization across the week due to repeated exposures

**Fig. 4:**
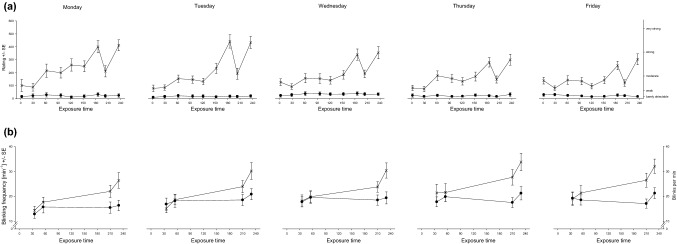
Eye irritation rating (**a**) and eye blinking frequencies (**b**) regarding across-days factor (5 exposure days: different diagrams), within-day factor (different measures), and condition/concentration factor (control condition: black circle and experimental condition: thin cross)

These analyses focused on *eye irritation* rating and eye blinking frequencies as both appeared to be the most sensitive indicators for sensory irritation due to controlled ethyl acrylate exposure (Kleinbeck et al. 2017). The cross-week effects were described for both ratings and eye blinking frequency, in parallel. Figure 4 shows the time courses of the reported eye irritation (Fig. 4a) and the eye blinking frequencies (Fig. 4b) for the two exposure conditions from Monday to Friday.

Statistically, the crucial carryover effects are indicated by significant three- or fourfold interactions of the factors *condition*, *day*, and *time* (*exposure duration* and *current concentration* for the eye blinking frequency). With respect to the ratings of eye irritation intensities, this threefold interaction becomes significant (*F*_32,862_ = 2.6, *p* < 0.001). Not only the two exposure conditions differed significantly, but also the time courses observed across the five subsequent test days showed some significant differences. In contrast, for the eye blinking frequencies the crucial fourfold interaction was not significant (*F*_12,240_ = 0.7, *p* = 0.8).

Figure 4a indicates that the crucial threefold interaction is caused by a different within-day time course on Mondays. While during the last peak exposure (see Fig. 1) almost no differences occurred between the 5 days, there was a stronger increase of the reported eye irritations during the first 2.5 h on Mondays compared to the other days. Overall, the reported eye irritation seems to decline across the week. In contrast, Fig. 4b shows that for the eye blinking frequency, the within-day time courses across the 5 days were highly comparable, but for all exposure conditions there seems to be a general increase of this physiological measure. Thus, this physiological readout indicates no effect accumulation after repeated exposures.

When collapsing the within-day measures of the eye blinking frequency and the eye irritation ratings, the different trends across the repeated exposures became more evident.

**Fig. 5:**
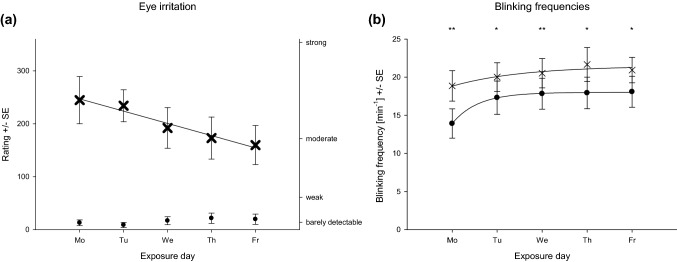
Eye irritation rating (**a**) and eye blinking frequencies (**b**) regarding across-days factor (5 exposure days) and condition/concentration factor (control condition: black circle and experimental condition: thin cross). Bonferroni corrected post hoc tests **p < 0.01 *p < 0.05

After aggregating the within-day measures of the rated eye irritation, Fig. 5a shows the significant interaction of the exposure condition with the five exposure days (*F*_4,112_ = 6.0, *p* < 0.001). During the 5 days, the subjective intensity decreased in the 0–10 ppm condition from clearly above to slightly below *moderate.* No clear trend could be observed in the 0 ppm condition. However, at the end of the exposure week, the inter-individual variance increases. Figure 5b shows that these across-week trends were not observed in the physiological measure of eye irritation. Here, the overall eye blinking frequencies only increased slightly from Monday to Tuesday. Nevertheless, on any single day, the difference between the control and the 0–10 ppm condition was significant (see Fig. 5b). This slight increase in eye blinking, however, is accompanied by an increase in rating from *barely detectable* (control condition) to around *moderate* (experimental condition; cf. Fig. 5a).

Generally, cross-week trends of these two end points (see Fig. 5) were best fitted by a decreasing linear function for the experimental condition in *eye irritation* rating (adj*R*^2^ = 0.94) and an exponential rise to maximum for eye blinking frequencies for control (adj*R*^2^ = 0.99) and experimental condition (adj*R*^2^ = 0.77). Nevertheless, both trends do not indicate sensitization across week.

### Impact of exposure peaks during repeated exposures

**Fig. 6:**
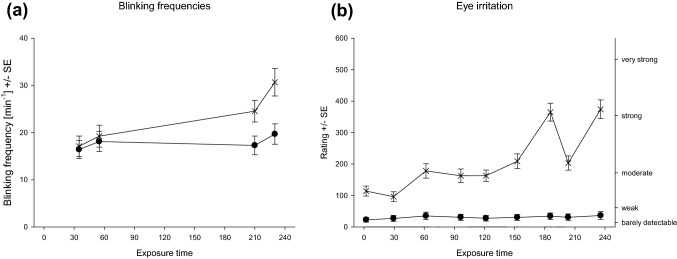
Eye blinking frequencies (**a**) and eye irritation ratings (**b**) regarding within-days factor (different measures) and condition/concentration factor (control condition: black circle and experimental condition: thin cross)

As already shown in Fig. 4, the applied exposure scenario yielded a prototypic temporal pattern on each day. This pattern was also observed and statistically modeled in the previous study investigating dose dependencies of sensory irritation caused by ethyl acrylate (Kleinbeck et al. 2017). When averaging across days, there was no marked impact of the first three exposure peaks (at exposure time of 0 min, 60 min, and 120 min) on eye irritation ratings and eye blinking frequency (see Fig. 6).

For eye blinking frequencies (Fig. 6a), there was a significant difference between the first Mackworth-clock test (starting at min 35) and the second Mackworth-clock test (starting at min 210; *F*_1,20_ = 29.1, *p* < 0.001) with higher eye blinking frequencies during the second measure. Additionally, the exposure peak of 10 ppm at that time point increased the average eye blinking frequency from 23 to 29 blinks/min. This increase was almost twice as high as the increases elicited by the first exposure peak or simply by performing the vigilance task. This distinct pattern was statistically confirmed by a threefold interaction of the factors condition, exposure time, and current concentration (*F*_1,20_ = 6.5, *p* < 0.05).

In Fig. 6b, the significant interaction of exposure condition and duration for eye irritation is given (*F*_8,224_ = 51, *p* < 0.001). In line with the eye blinking frequency results, strong eye irritations (Fig. 6b) were only reported during the last exposure peaks. Such a distinct effect of the *current concentration* could not be observed during the first exposure peak of the 0–10 ppm condition (exposure time 0, 30, and 60 min). Bonferroni corrected post hoc test in the 0–10 ppm condition revealed a stepwise increase of the reported eye irritation. While the first two ratings did not differ from each other, they were significantly lower than all ratings obtained at later time points. The next plateau of moderate eye irritations with non-significantly different ratings lasted from 60 to 150 min. During the third phase (180–240 min), the current concentration is clearly mirrored in the ratings of the volunteers. During the 10 ppm peaks (180 and 240 min), strong eye irritations were reported that significantly differed from any other time point.

**Fig. 7:**
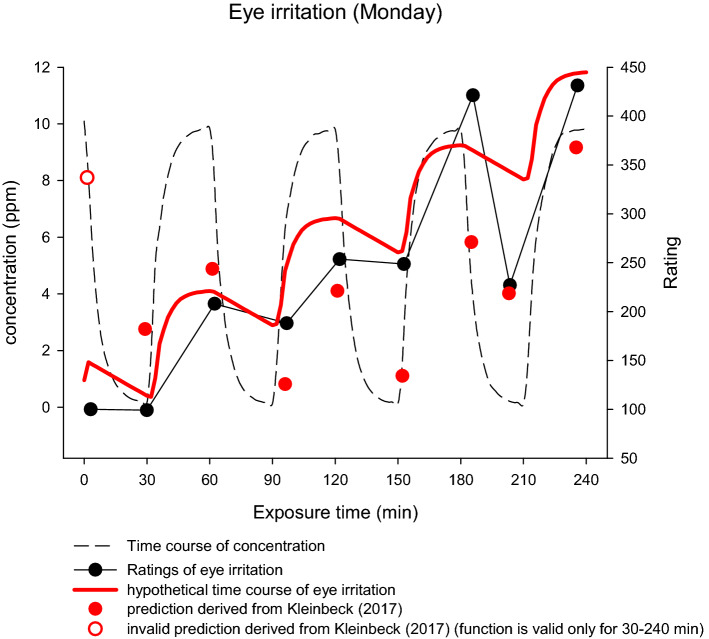
Hypothetical time course of eye irritation based on assumptions derived from PK/PD models of the rabbit eye (red line) in relation to the time course of concentration (dashed line) and the ratings of eye irritation at different time points at day 1 of exposure (black dots) together with predicted values (red dots) derived from a model fitted to the data of Kleinbeck et al. (2017) (colour figure online)

This distinct pattern of a combined effect of exposure duration and concentration was only observed for the reported eye irritation, a perceptual indicator of sensory irritation. Most of the other olfactory and trigeminal mediated perceptions were influenced more strongly by the current concentration. In Kleinbeck et al. (2017), the complex function ($f\left(t\right)={Y}_{0}+a\times t+b\times {t}^{2}+c\times \mathrm{sin}(\frac{2\pi \times t}{60}+1.5)$) fitted to eye irritation ratings regarding the exposure time and current concentration yielded an excellent fit (adj*R*^2^ = 0.92). However, the predicted ratings from this function, even with an adjusted level for this study, do not match well with the factual ratings showing the largest differences during the last hour of exposure (cf. Fig. 7; red vs. black dots). As sensory irritation seems to be more persistent than odors, the influence of current concentration might be modeled in a different manner. Irritation of the eye caused by a local irritant is detected by sensory nerve terminals in the cornea (target site). It is reasonable to assume that the higher the concentration at the target site, the higher is the perceived irritation. A gaseous substance is dissolved in the tear fluid where clearance processes take place. Pharmacokinetic and pharmacodynamic (PK/PD) models describe such processes of accumulation and clearance at target organs. Sakanaka et al. (2008) demonstrated—common to the investigated target sites (cornea, aqueous humor, and iris–ciliary body) of the rabbit eye—a steep increase of timolol concentrations after instillation of a rise-to-maximum shape (a few minutes at the cornea up to about 30 min at the iris–ciliary body). This increase is followed by a less steep linear decrease, leading to baseline in about 120 min. Using such an increase–decrease time course, irritation at the cornea could be roughly modeled (cf. Fig. 7; red line) by mimicking a steep accumulation (similar to the concentration increase) and a less steep linear decrease of ethyl acrylate concentration at the cornea.

The non-perfect clearance (as a consequence of lack of time) might lead to an accumulation of the irritant impacting the eye. Even this coarse model fits well with the rated eye irritation on Monday (cf. black dots in Fig. 7). Two deviating ratings (122 min/fifth rating and 203 min/eighth rating) were conducted during breaks without visual demands (except the first rating, all other ratings were preceded by tests with visual demands: two-back task, Mackworth-clock task, divided attention task, flanker task). As eyes could be closed voluntarily and kept closed for longer time periods during breaks, perceived eye irritation might be lower. The same temporal pattern of ratings is seen on the other exposure days (cf. Fig. 4a) and, therefore, the suggested model suits well the ratings on Tuesday to Friday and to the mean ratings summarizing all days (cf. Fig. 6b), indicating again a high reliability of the rating method. For *burning* ratings, a similar temporal pattern of ratings is observed as in *eye irritation* ratings. As only the perception of *burning* without specification of target organ (eyes or nose) was rated, it remains unclear whether the same temporal pattern is due to *burning* perception in the eye or in the nose.

### Effects of repeated exposures on markers of neurogenic inflammation/inflammation and nasal congestion

There were no significant differences between the concentration/conditions and days in substance *P* concentrations (*χ*^2^_9_ = 11.8, *p* = 0.22). This lack of neurogenic inflammation due to the experimental exposures to ethyl acrylate also leads to a lack of any “immune response”. There were neither significant differences between the concentration/conditions and days in TNF-α nor in interleukin-8 concentrations.

Functional effects on nasal breathing could neither be confirmed on a single day, nor as a result of the repeated exposures to the local irritant. There was no impact of concentration/condition on nasal congestion as measured by active anterior rhinomanometry (*F*_1,12_ = 0.928, *p* = 0.35). Due to technical reasons and colds in some subjects during the exposure days, the data of only 13 subjects were complete and could be analyzed.

### Effects of repeated exposures on behavior

**Fig. 8:**
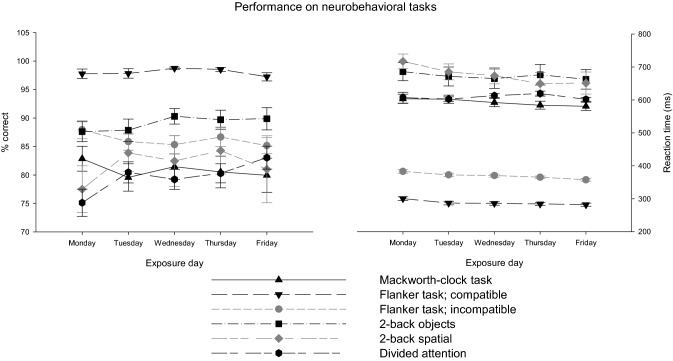
Change in performance (% correct and correct responses’ reaction time) for the investigated neurobehavioral tests (flanker task is split in performance on compatible and incompatible stimuli)

There was no significant influence of ethyl acrylate concentration on the performance of any neurobevahioral task. However, regarding the across-days factor, there are signs of change from day to day in each task (Fig. 8).

There is a significant impact of days on correct responses for the Mackworth-clock test and the divided attention task (*F*_4,116_ = 5.2, *p* < 0.001 and *F*_4,112_ = 4.9, *p* < 0.001). Post hoc tests reveal only a significant decrease in correct responses from Monday to Tuesday in Mackworth-clock test and a significant increase from Monday to Tuesday and Friday for the divided attention task. There is a significant impact of days on reaction times for both variants of the flanker task (compatible: *F*_4,108_ = 12.7, *p* < 0.001 and incompatible: *F*_4,108_ = 15.3, *p* < 0.001) and the spatial two-back task (*F*_4,36_ = 3.7, *p* < 0.05; due to technical reasons the complete data of only 10 subjects were available). Post hoc tests reveal that reaction time in the compatible flanker task is slower on Monday compared to any other day. The same is true for the incompatible variant of the flanker task where, additionally, reaction time on Friday was faster than that on any other day. No differences between days could be revealed by post hoc tests in the spatial two-back task.

All in all, there is only one sporadic deterioration on performance, while performance mainly improves or stays the same from day to day independent of ethyl acrylate exposure.

## Discussion

In this short-term inhalation study, comparable to recent developments in animal research, subjects were exposed to a varying concentration of ethyl acrylate on five subsequent days to ensure time extrapolations in the case of human sensory irritation and to evaluate possible carryover effects in humans. This exposure scenario exploits the maximal amount of peak exposures per work shift (DFG 2018) and, thus, with respect to the end point sensory irritation a worst-case concentration profile was tested. As outlined in the introduction, ethyl acrylate serves as an excellent model compound with a good database of relevant human and animal studies. To our knowledge, this is the first study doing such an evaluation in a realistic work setting.

### Replication of previous results

Higher eye blinking frequencies were observed during the experimental condition (with exposure) compared to the control condition. This result replicates the results of a previous study on ethyl acrylate (Kleinbeck et al. 2017). Moreover, a recent study investigating the effects of comparable ethyl acrylate exposures in a sample including atopic subjects (Sucker et al. 2019) also showed that exposures to 5 ppm significantly increased the eye blinking frequency. A similar effect could be demonstrated for 2-ethylhexanol (Kiesswetter et al. 2005). For other compounds such as ammonia, this physiological marker of sensory irritation was not increased during experimental exposures in the range of permitted OELs (Pacharra et al. 2017).

The same effect of experimental condition is seen in ratings of *eye irritation*. This correspondence between ratings and physiological response has also been shown before (Kleinbeck et al. 2008, 2017; Walker et al. 2001). However, mathematical modeling of the association between perception and physiology is difficult and might partly depend on high responders (Ernstgard and Bottai 2012) and sufficiently high exposure conditions representing human LOAECs (Kiesswetter et al. 2005).

Differences in the profiles of the LMS ratings, when compared to Kleinbeck et al. (2017), might be due to differences between the two independent samples or obvious differences in experimental design, such as different number of experimental conditions and, therefore, first experimental exposure to ethyl acrylate in different concentrations. At least for the LMS ratings *odor intensity* and *eye irritation*, the time courses repeatedly observed in our experiment were comparable to those given by Sucker et al. (2019). Thus, the validity of this assessment could be confirmed by within and across laboratory comparisons.

### Short- and long-term time courses

In contrast to Sucker et al. (2019), Kleinbeck et al. (2017) and other experimental exposure studies (e.g., Ernstgard et al. 2006; Ernstgard et al. 2012; Hey et al. 2009; Juran et al. 2014; Juran et al. 2012; Kleinbeck et al. 2008; van Thriel et al. 2006; van Thriel et al. 2010), we are now able to analyze short-term time courses within a day and more long-term time courses across a week. Here, the most striking observation is the clear stability of the within-day pattern of the eye irritation ratings and eye blinking frequencies across the 5 days. Nevertheless, there were some differences between the ratings and the physiological marker of eye irritation. While the eye blinking frequency pattern is the same on each day, the general level of the ratings declined across the 5 days. However, the concentration dependency of the ratings at the end of the 4-h exposure remains a unique feature of this pattern. Despite the decreasing trend observed for the average ratings, the last ratings given during exposure peaks indicate strong eye irritations on all 5 days of the experiment.

The design of our study would be capable of detecting carryover or sensitization effects across a week of repeated exposures. However, neither the biochemical analyses of the nasal lavage fluid, nor the functional measures of nasal breathing were affected by repeated exposures to ethyl acrylate that reproducibly induced signs of adverse sensory irritation in humans (Brüning et al. 2014).

A marked increase in *eye irritation* ratings could only be observed during the last hour of exposure on every exposure day. This was also shown by Sucker et al. (2019). More physiology based, this might be caused by a temporal accumulation of ethyl acrylate on the surface of the outer eye. Accordingly, we propose a model for the time course of eye irritation ratings that mimics the processes of accumulation and clearance derived from a PK/PD model of the rabbit eye (Sakanaka et al. 2008). This suggested model fits to the ratings and, therefore, is interpreted as a hint of the validity of the rating method and the physiological processes underlying sensory irritation in general. To clarify the relation between processes at the eye and perceptions of eye irritation, more research is needed with respect to accumulation and clearance of local irritants in the tear fluid and their subsequent effects on epithelial and neuronal structure of the eye. Furthermore, at higher concentrations of an irritant, additional and modulating processes related to the immune system might cause sensitization. Our results of the biochemical parameters did not reveal any hints for neurogenic or immune cell-related inflammatory signaling (Chiu et al. 2012; Hunter et al. 2000; Nikasinovic-Fournier et al. 2002). Furthermore, no carryover effects from day to day could be found for both, reported eye irritation and recorded eye blinking frequency.

Especially, the perception of malodors has been assumed to cause behavioral alteration due to distractive effects in experimental exposure studies (Brüning et al. 2014; Hey et al. 2009; van Thriel et al. 2003, 2007b). The performance in various neurobehavioral tasks that differ with respect to the cognitive function that they are thought to assess was independent of ethyl acrylate exposure. The performance (mainly reaction time) improved over the consecutive days in most of the tasks. Therefore, also the learning curve is not disturbed by ethyl acrylate exposure that repeatedly leads to slight sensory irritation at the eye on subsequent days.

### General discussion

No accumulating effects of a varying concentration exposure to ethyl acrylate could be observed on five subsequent days, neither in subjective ratings, physiological measures, nor in neurobehavioral tasks when exposing volunteers to concentrations permitted by the OEL in Germany that was valid at the time of the experiment (5 ppm with a short term exposure level of 10 ppm). Regarding eye irritation across week, a slight divergence of subjective and objective measures could be demonstrated. The decrease in eye irritation ratings might be due to general habituation effects to the experimental procedure that might be more pronounced on the perceptual level (ratings and symptoms) than on the physiological level (eye blinking frequency). Nevertheless, when taking into account plausible assumptions derived from PK/PD modeling of the rabbit eye (Sakanaka et al. 2008), the non-linear time course of the perceived eye irritation could be explained. While perceptual ratings were often regarded as ‘soft’ methods (Arts et al. 2006; Paustenbach et al. 1997; Philpott et al. 2006) as they combine sensory and cognitive odor processing (van Thriel et al. 2008), these results clearly showed that these measures were valid and reliable tools for the assessment of sensory irritation in humans.

In well-controlled, experimental exposure studies, the standardized and repeated assessment of the eye blinking frequency can be regarded as a valid and sensitive method for measuring sensory irritation (cf. Kiesswetter et al. 2005; Kleinbeck et al. 2017). The current study adds further support to this assumption by showing that a unique response pattern could be found on any single day of the five subsequent exposures.

At least for the irritant ethyl acrylate, the other physiological and biochemical markers were not suitable to detect exposure-related differences. The biochemical markers in nasal lavage fluid (NALF) were selected according to the response pathways suggested by Brüning et al. (2014). After the stimulation of peripheral nerve fibers by a sensory irritant, substance *P* can be secreted from axonal vesicles into the surrounding tissue (Chiu et al. 2012). Here, the neuropeptide exhibits immunomodulatory effects by activating dendritic cell/macrophages as well as lymphocytes that subsequently release various cytokines (Mashaghi et al. 2016). Among them are the two acute phase cytokines interleukin-8 (IL-8) and tumor necrosis factor alpha (TNFα) that were measured in the NALF of the volunteers. Even though the eye blinking frequency was significantly increased and strong burning and pungent sensations were reported especially at the end of the ethyl acrylate exposures, the activation of intranasal trigeminal nerve fibers seems to be insufficient to elicit the release of substance *P*. Thereby, the subsequent signaling from immune or other surrounding cells (e.g., epithelial cells) seems to be prevented. That is, with the activation of the trigeminal nerve, we identified a key event of the adverse outcome pathway described by Martinez and Eling (2019; key event 2: trigeminal acitivation), but as there is no hint for neurogenic inflammation (key event 3), thus, the adverse outcome pathway seems to be interrupted at this point. With these results, we could (a) replicate previous findings (Kleinbeck et al. 2017) and (b) provide first evidence that the repeated induction of slight sensory irritation does not cause any sensitization of neurogenic reflexes across a working week. However, repeated exposures to other, structurally unrelated sensory irritants have to be investigated experimentally or epidemiologically to draw more reliable conclusions.

The ocular surface is supposed to be among the most densely innervated structures of the human body (Beuerman and Stern 2005; Moreira et al. 2007). Recently, substance P in tears has been suggested as a potential biomarker for contact lens (CL) discomfort, as it was significantly elevated in symptomatic CL wearers (Lopez-de la Rosa et al. 2019). Thus, the biochemical analyses of tears might be more sensitive and maybe stronger linked to the observed increase of the eye blinking frequency. However, the collection of tears is more invasive (Zhou and Beuerman 2012) and might cause irritation by itself.

In addition to the avoidance of sensory irritation, odor effects such as impaired task performance due to distractive effects of malodors (van Thriel et al. 2007b) have been discussed as indicators of “adverse odor effects”. The behavioral tasks applied in this study did not reveal any impairment of cognitive function. This is in line with the findings reported by Sucker et al. (2019) using a demanding working memory task to assess cognitive functioning during ethyl acrylate exposures. Several issues related to the olfactory processing of this sensory irritant might hamper the induction of distractive effects. Ethyl acrylate has a low odor threshold (0.007 ppb) that is accompanied by a huge confidence interval, indicating large inter-individual differences (van Thriel et al. 2006). Thus, individual differences in the perceptibility of a particular odor and other non-sensory factors associated with odor processing described in previous research (van Thriel et al. 2008) impede the identification of “adverse odor effects” in experimental exposure studies.

### Relevance for integrative risk assessment

Mechanistic knowledge about the molecular initiating event (MIE) and related key events (KEs) can be derived from in vitro studies (Lehmann et al. 2016, 2017) or existing databases as proposed in the recent AOP paper (Martinez and Eling 2019). As a consequence, animal studies like the RD_50_ bioassay can be reduced by pre-selecting only relevant and highly potent compounds. Thereby, the current strategy in toxicology to reduce and replace animal testing (National Research Council 2007) can also be achieved for the end point sensory irritation. Moreover, the structured assembly of relevant data as proposed by the AOP (Martinez and Eling 2019) together with systematic read-across from data-rich compounds (e.g., ethyl acrylate) offers the opportunity to predict concentrations that might be in the range of human NOAECs. According to an integrated risk assessment strategy for the avoidance of sensory irritation, experimental exposure studies of human volunteers might be considered as a final stage of gathering toxicological data. Thereby, many uncertainties associated with animal or in vitro testing can be reduced. However, it must be clearly stated that this idea is only applicable in the context of sensory irritation.

### Limitations

Due to logistical constraints, we were only able to expose volunteers for 4 h/day on five consecutive days. Accordingly, the extrapolation of our results to the entire working life (8 h/day, 5 days/week for 40 years) possesses some uncertainties. Epidemiological studies using sensitive and innovative methods (e.g., biochemical analysis of NALF and tears) are needed to close the knowledge gap between experimental exposure studies with human volunteers and the real working environment.
